# AI in fungal drug development: opportunities, challenges, and future outlook

**DOI:** 10.3389/fcimb.2025.1610743

**Published:** 2025-05-21

**Authors:** Yanjian Li, Yue Qiao, Yuanyuan Ma, Peng Xue, Chen Ding

**Affiliations:** ^1^ College of Life and Health Sciences, Northeastern University, Shenyang, China; ^2^ School of Public Health, Nantong University, Nantong, China

**Keywords:** fungal infection, artificial intelligence, antifungal resistance, drug discovery, machine learning

## Abstract

The application of artificial intelligence (AI) in fungal drug development offers innovative strategies to address the escalating threat of fungal infections and the challenge of antifungal resistance. This review evaluates the current landscape of fungal infections, highlights the limitations of existing antifungal therapies, and examines the transformative potential of AI in drug discovery and development. We specifically focus on how AI can enhance the identification of new antifungal agents and improve therapeutic strategies. Despite numerous opportunities for advancement, significant challenges remain, particularly regarding data quality, regulatory frameworks, and the complexities associated with the drug development process. This review aims to provide insights into recent advancements in AI technologies, their implications for the future of fungal drug development, and the necessary research directions to effectively leverage AI for improved patient outcomes.

## 1 Introduction

Fungal pathogens represent a significant threat to global health, with a notable increase in the incidence of invasive fungal diseases (Xu, 2022; Denning, 2024). Despite their profound impact, human fungal infections remain inadequately studied (Denning and Bromley, 2015). The intricate mechanisms underlying their pathogenicity, combined with a limited range of antifungal therapies, present considerable challenges for treatment (Brown et al., 2012a, 2012b; Lockhart et al., 2023). Current scientific efforts are focused on developing more effective antifungal agents, advancing vaccine research, and gaining a deeper understanding of fungal pathogenicity (Brown et al., 2012a, 2012b; Bongomin et al., 2017; Limper et al., 2017). In October 2022, the World Health Organization (WHO) released its first list of priority fungal pathogens, categorizing 19 species into three urgency tiers: critical, high, and medium. The critical tier includes pathogens *Cryptococcus neoformans*, *Candida auris*, *Aspergillus fumigatus*, and *Candida albicans*, while the high-priority tier encompasses *Candida glabrata*, *Histoplasma* species, *Mucorales*, and others. The medium-priority tier includes pathogens like *Coccidioides* species and *Pneumocystis jirovecii* (WHO, 2022). Recent estimates indicate that approximately 6.5 million individuals are affected by invasive fungal infections annually, resulting in around 3.7 million deaths. This figure represents a concerning increase from earlier estimates, which ranged from 1.5 to 2.0 million fatalities per year (Brown et al., 2024; Denning, 2024). The rising prevalence of antifungal resistance complicates the treatment of these infections, leading to increased morbidity and mortality rates (Fisher et al., 2022; Lockhart et al., 2023; Ma et al., 2025). WHO reports indicate a troubling trend in invasive fungal infections, which manifest as severe systemic conditions such as candidiasis, aspergillosis, and cryptococcosis (WHO, 2022). These infections not only prolong hospital stays but also impose significant economic strains on healthcare systems. Notably, species from the *Candida* and *Aspergillus* genera have demonstrated the ability to evade early detection and appropriate treatment protocols, complicating clinical management. For example, while *C. albicans* is typically a benign commensal organism within the human microbiome, it can transition to a pathogenic state under certain conditions (Li et al., 2022). Similarly, *A. fumigatus*, which thrives in soil and decaying organic matter, poses a risk of life-threatening pulmonary infections in vulnerable individuals (Arastehfar et al., 2021; Earle et al., 2023). A critical issue in managing fungal infections is the emergence of antifungal resistance, which has only recently received comparable attention to antibiotic resistance. The mechanisms of resistance vary and can differ significantly among fungal species; notably, *C. auris* has emerged as a multidrug-resistant yeast that presents a serious public health challenge due to its resistance to multiple classes of antifungal agents (Jacobs et al., 2022). The therapeutic landscape for treating fungal infections is primarily restricted to antifungal drugs (Shirsat et al., 2021; Carmo et al., 2023; Roy et al., 2023). While these medications can be effective against specific pathogens, their use is often constrained by factors such as toxicity, narrow spectrum of activity, and the potential for drug-drug interactions (Nett and Andes, 2011; Corrêa-Junior et al., 2025). Furthermore, the scarcity of adequate treatment options for polymicrobial infections exacerbates the challenges faced in managing fungal diseases.

In light of the urgent need for innovative drug discovery and development strategies, artificial intelligence (AI) has emerged as a promising technology that could facilitate the swift identification and development of novel antifungal agents. By leveraging machine learning and deep learning, AI can process extensive datasets, uncover hidden patterns, and identify potential drug candidates at unprecedented rates (Farghali et al., 2021; Abbas et al., 2024; Sharma et al., 2025). For instance, research by Li et al. has demonstrated that machine learning classifiers can effectively identify and rank drug resistance mutations in *C. auris*. New mutations such as R278H in the *ERG10* gene and I466M and Y501H in the *ERG11* gene, alongside known resistance mutations, may contribute to fluconazole, amphotericin B, and micafungin resistance. This advancement enhances our understanding of the resistance mechanisms of this pathogen and provides a cost-effective approach to analyzing drug resistance (Li et al., 2021). Moreover, a study led by Yeji Wang and colleagues presented an AI-driven latent diffusion model capable of generating a diverse array of effective antimicrobial peptides (AMPs). This model addresses several limitations of current methods in producing AMPs with sufficient novelty and diversity. Notably, their approach resulted in 25 out of 40 synthesized peptides exhibiting antifungal properties. Among these, AMP-29 showed specific antifungal activity against *C. glabrata* and proved effective in an *in vivo* murine skin infection model (Wang et al., 2025). The incorporation of AI into fungal drug development has the potential to deepen our understanding of fungal biology and resistance mechanisms, leading to more precise and effective treatment strategies. Additionally, AI could enable the personalization of antifungal therapies by integrating patient-specific information, thereby enhancing treatment outcomes and reducing adverse effects. The incorporation of AI into fungal drug development constitutes a promising strategy to confront the urgent challenges posed by antifungal resistance and the limitations of existing therapies. By harnessing advanced technologies, researchers can expedite the discovery of new antifungal agents and foster personalized therapeutic approaches, ultimately improving patient outcomes and addressing the increasing threat of fungal diseases. This review highlights the pressing need for enhanced antifungal strategies, examines the potential opportunities presented by AI-driven approaches in the development of antifungal drugs, and analyzes the challenges associated with integrating AI into this crucial field.

## 2 The urgent need for enhanced antifungal strategies

The rising incidence of invasive fungal infections highlights an urgent requirement for the development and implementation of enhanced antifungal strategies (Stewart and Paterson, 2021; Mudenda, 2024). On April 1, 2025, the WHO released its inaugural report on the detection and treatment of fungal infections, which illuminated the critical shortage of effective drugs and diagnostic tools available to combat these conditions. The current repertoire of antifungal medications is insufficient to address the increasing prevalence and complexity of these infections. The obstacles encountered in the treatment of fungal diseases are numerous and multifaceted, including diagnostic delays, limited therapeutic options, and the emergence of resistant strains.

### 2.1 Diagnostic challenges

Timely and accurate diagnosis is essential for the effective management of invasive fungal diseases. Traditional diagnostic methods, such as culture techniques and histopathological examinations, often prove to be time-consuming and may lack the sensitivity required for certain pathogens (Fang et al., 2023). For instance, fungal cultures can take several days to yield results, leading to delays in initiating critical treatment. Additionally, histopathological assessments can be limited by the necessity for invasive procedures and the availability of specialized expertise, which is not always accessible (Fang et al., 2023). To address these diagnostic challenges, it is crucial to innovate and enhance diagnostic tools that offer rapid and precise identification of fungal infections. Recent advancements in molecular diagnostic techniques, including polymerase chain reaction (PCR)-based methods and next-generation sequencing (NGS), have demonstrated significant promise for the swift detection and identification of fungal pathogens, even in low-burden settings (Babady et al., 2024; Pham et al., 2024). Moreover, changes in the gut microbiome may be associated with fungal infections, such as *Cryptococcus*, and analyzing these alterations could assist in diagnosing cryptococcal meningitis. Thus, gut microbiome testing presents a potentially valuable approach for detecting fungal infections, although further research is required to validate its effectiveness (Ma et al., 2023). Integrating these advanced technologies into clinical practice could greatly enhance diagnostic accuracy and speed, facilitating the timely initiation of targeted antifungal therapies. Furthermore, the application of machine learning algorithms in analyzing clinical data and imaging could further enrich the diagnostic landscape, enabling more personalized treatment strategies.

### 2.2 Limitations of current antifungal therapies

Current antifungal agents are categorized into six main classes: azoles, polyenes, echinocandins, pyrimidine analogs, mitotic inhibitors, and allylamines (**Table 1**) (Houšť et al., 2020; Shirsat et al., 2021). While these drugs are effective against specific fungal pathogens, several inherent limitations hinder their overall efficacy. One significant challenge is the emergence of antifungal resistance, particularly among common pathogens such as *Candida* and *Aspergillus* species (Goncalves et al., 2016; Perlin et al., 2017). Resistance mechanisms include alterations in drug targets, overexpression of efflux pumps, and biofilm formation, all of which threaten the effectiveness of existing therapies (Morace et al., 2014; Goncalves et al., 2016; Lee et al., 2021). The rising prevalence of resistance underscores the necessity for continuous surveillance and the implementation of antifungal stewardship programs aimed at mitigating resistance development. Additionally, many antifungal agents exhibit a limited spectrum of activity, restricting their utility in treating polymicrobial infections (Wiederhold, 2022; Roy et al., 2023). When fungal infections are caused by multiple species, reliance on a single agent can lead to treatment failures. This limitation emphasizes the need for exploring combination therapy strategies that broaden the spectrum of efficacy while minimizing the risk of resistance. Additionally, some antifungal medications are notable for their significant toxicity, necessitating careful patient monitoring and dose adjustments. For example, azoles have been linked to hepatotoxicity, requiring clinicians to balance therapeutic efficacy with potential adverse effects. Azoles like fluconazole are commonly prescribed for candidiasis; however, their effectiveness can be compromised by the emergence of resistance and associated hepatotoxicity (Daneshnia et al., 2023; Rakhshan et al., 2023; Sobel et al., 2023). Amphotericin B, another widely used antifungal, is also known for its toxicity (Cavassin et al., 2021). It can cause nephrotoxicity, which often leads to reduced renal function and electrolyte imbalances, particularly hypokalemia. These adverse effects necessitate meticulous monitoring of kidney function and electrolyte levels during treatment (Laniado-Laborín and Cabrales-Vargas, 2009; Karunarathna et al., 2024). Due to its potential for toxicity, the use of amphotericin B typically requires careful consideration of the risk-to-benefit ratio, especially in patients who may already have compromised renal function or other underlying health issues. Despite its significant efficacy against serious fungal infections, the associated toxicities limit its use and highlight the need for alternative therapies or adjunctive strategies to mitigate these risks. Given these considerable limitations, there is an urgent need for innovative approaches in antifungal drug development. Historically, the pharmaceutical industry has prioritized antibiotic development over antifungals, leading to a relative lack of investment and research in this critical area. To address this gap, exploring novel mechanisms of action, repurposing existing drugs, and investing in the development of new antifungal classes is essential. Additionally, fostering collaborations between academia, industry, and healthcare providers can accelerate the translation of research findings into clinical practice, ultimately enhancing patient outcomes in the fight against invasive fungal infections.

**Table 1:** Main antifungal drug classes, common drugs, their mechanisms of action, and limitations.

Antifungal class	Common drugs	Mechanism of action	Limitations
Polyenes	Amphotericin B	Binds to sterols in fungal membranes, forming pores	Nephrotoxicity, limited spectrum
Azoles	Fluconazole, Itraconazole	Inhibit sterol synthesis in fungal membranes	Resistance, hepatotoxicity
Echinocandins	Caspofungin, Micafungin	Inhibit fungal cell wall synthesis	Limited activity against some fungi
Pyrimidine analogs	Flucytosine	Inhibit RNA and DNA synthesis	Resistance when used alone, bone marrow suppression
Mitotic inhibitors	Griseofulvin	Interfere with fungal cell division	Limited use, mainly for dermatophytes
Allylamines	Terbinafine	Inhibit fungal membrane biosynthesis	Mainly for dermatophytes, liver interactions

## 3 AI: a transformative force in drug development

The integration of AI into drug development represents a paradigm shift in the way new antifungal agents are discovered and optimized. By harnessing advanced technologies such as machine learning, deep learning, natural language processing (NLP), reinforcement learning and generative adversarial networks (GANs), AI provides researchers with powerful tools for analyzing complex biological datasets, predicting drug interactions, and refining lead compounds more effectively than traditional methods (Abbas et al., 2024; Zhang et al., 2025). Each of these technologies offers unique advantages at different stages of drug development, which will be elaborated upon in the following sections.

### 3.1 Enhanced drug discovery

AI inherent ability to process vast volumes of biological and chemical data significantly enhances the efficiency of the drug discovery process (Zhang et al., 2025). Traditional methodologies for drug discovery often involve labor-intensive and time-consuming high-throughput screening approaches, which can be limited by their capacity to discover novel compounds. In contrast, machine learning algorithms can rapidly analyze extensive datasets that include genomic, proteomic, and metabolomic information. For instance, machine learning models trained to recognize patterns can identify intricate correlations that may elude conventional analytical approaches. Specifically, these models can analyze genomic sequences to pinpoint genetic variations associated with disease states, facilitating the identification of potential drug targets (Paul et al., 2024). Deep learning methods, which excel in feature extraction from large datasets, enable faster and more accurate identification of promising drug candidates. Additionally, NLP techniques can mine vast amounts of scientific literature, extracting relevant information that informs drug discovery efforts. This accelerated identification process not only shortens the drug discovery timeline but also enhances the likelihood of success in developing effective therapies (Zhang et al., 2025). Moreover, predictive modeling capabilities, particularly through machine learning, are instrumental in assessing potential drug interactions and toxicity profiles (Amorim et al., 2024). By leveraging historical data from previous pharmacological studies, these models can forecast how new compounds might behave within biological systems, crucial for identifying viable drug candidates while mitigating adverse effects. For instance, machine learning algorithms can predict pharmacokinetic properties, such as absorption and metabolism rates, based on the chemical structure of compounds, informing key decisions in the drug development process (Chou and Lin, 2023; Tran et al., 2023).

### 3.2 Drug repurposing and optimization

In addition to novel drug discovery, AI significantly enhances drug repurposing strategies (Ashiwaju et al., 2023; Singh, 2024). Drug repurposing involves identifying new therapeutic applications for existing compounds, which can substantially reduce the time and financial resources associated with developing new therapies. Given that the safety profiles and pharmacological properties of established drugs are already well-characterized, AI-driven platforms can analyze extensive clinical datasets and relevant literature—using NLP techniques—to unveil potential new indications for existing antifungal agents. This acceleration in the repurposing process is particularly valuable in urgent public health challenges, where rapidly discovering effective treatments for diseases with limited therapeutic options is paramount. Once promising candidates are identified, AI can further aid in optimizing these lead compounds. Advanced techniques such as reinforcement learning and GANs can be employed to design novel chemical structures that exhibit desirable biological properties (Wang et al., 2017; Popova and Isayev, 2018). By simulating biological responses to various chemical modifications, these innovative approaches streamline the optimization process and foster creativity in drug design, leading to potentially groundbreaking therapeutic interventions.

### 3.3 Predictive modeling in clinical trials

AI transformative impact extends into clinical trial design and patient selection (Pencina and Peterson, 2016). By analyzing real-world patient data and demographics, AI systems can identify the most suitable populations for clinical trials, improving recruitment efficiency and increasing the chances of successful trial outcomes (Köpcke et al., 2013). This targeted approach ensures that clinical trials are representative of the broader patient population, enhancing the generalizability of findings. Furthermore, AI can predict patient responses to specific treatments by analyzing genetic and phenotypic data, facilitating the development of personalized medicine approaches. This capability aligns with the increasing emphasis on precision medicine, where treatment strategies are tailored to the unique characteristics of individual patients. For instance, machine learning techniques can analyze datasets to identify biomarkers that correlate with treatment efficacy or toxicity, allowing for a more individualized approach to therapy. By leveraging AI in this manner, researchers can optimize treatment efficacy and minimize adverse reactions, ultimately leading to improved patient outcomes.

The integration of AI into drug development is not merely a trend but a transformative force that holds the potential to reshape the landscape of therapeutic discovery and optimization. By streamlining the drug discovery process, facilitating drug repurposing, and enhancing clinical trial design, AI empowers researchers to develop more effective and safer treatments at an accelerated pace. As the field continues to evolve, the collaboration between AI technologies and traditional pharmacological practices will undoubtedly pave the way for significant advancements in healthcare.

## 4 Opportunities in AI-driven fungal drug development

Integrating AI into fungal drug development presents several key opportunities to effectively address the challenges posed by fungal infections and resistance mechanisms.

### 4.1 Accelerated drug discovery

AI has the potential to significantly expedite the identification of novel antifungal compounds by analyzing chemical libraries and predicting their biological activity. Machine learning algorithms can process vast amounts of biological and chemical data, uncovering promising candidates that may be overlooked in traditional screening processes. For instance, AI can identify novel compounds targeting specific fungal mechanisms, such as enzymes involved in cell wall synthesis or critical metabolic pathways. By creating accurate predictive models, researchers can rapidly screen for potential compounds and optimize drug structures based on predictive results, thus accelerating the overall drug development process.

### 4.2 Predictive modeling for efficacy and safety

The predictive capabilities of machine learning algorithms can significantly reduce the time and costs associated with clinical trials by estimating the efficacy and safety of drug candidates (Kolluri et al., 2022). By leveraging existing datasets and employing predictive analytics, researchers can prioritize the most promising compounds for further development. This capability is crucial for the early identification of potential side effects or adverse reactions, enabling informed decisions about which candidates to advance. Through comprehensive analysis of clinical data and compound characteristics, AI can help build more accurate models for predicting drug safety and efficacy, enhancing the likelihood of clinical trial success.

### 4.3 Personalized medicine approaches

AI can play a pivotal role in tailoring antifungal treatments based on individual patient profiles, improving treatment outcomes and minimizing adverse effects. By analyzing patient-specific data, such as genomic information, microbiome composition, and prior treatment responses, AI empowers clinicians to make more informed decisions regarding antifungal therapies. Personalized medicine approaches have the potential to lead to more effective treatments and improved patient adherence to therapy. Furthermore, AI can facilitate real-time monitoring of patient responses to treatment, allowing for timely adjustments to therapy to achieve optimal outcomes.

### 4.4 Integration of multi-omics data

One of AI greatest strengths lies in its ability to integrate diverse datasets, including genomic, proteomic, metabolomic, and clinical data (Grapov et al., 2018). By leveraging multi-omics approaches, researchers can gain a holistic understanding of fungal pathogens and their interactions with host systems, paving the way for the development of targeted therapies. The combination of omics data with patient demographics and treatment outcomes can further enhance predictions regarding therapy efficacy and safety, enhancing the precision of treatment strategies. Additionally, AI can identify potential biomarkers that may exist within different patient populations, facilitating the development of tailored therapeutic strategies suited to specific groups.

## 5 Challenges in integrating AI into fungal drug development

Despite the exciting prospects of AI in drug development, several challenges hinder its successful integration into the field of fungal infections.

### 5.1 Data quality and availability

The effectiveness of AI models largely depends on the quality and availability of high-quality, annotated datasets. However, in fungal research, such datasets are often scarce. The absence of standardized data formats can introduce biases in AI predictions, limiting the generalizability and applicability of the developed models. To address these challenges, there is an urgent need to create comprehensive, well-annotated datasets that encompass a wide array of fungal species, resistance mechanisms, and patient responses. Collaborative efforts among researchers, academic institutions, and industry stakeholders are crucial for building robust datasets that can drive AI-powered innovations. Initiatives such as shared databases, open-access repositories, and standardized data collection protocols can facilitate the aggregation of high-quality data essential for training effective AI models.

### 5.2 Regulatory hurdles

The intricate nature of drug development and the variability of regulatory frameworks can significantly impede the adoption of AI-driven solutions. Regulatory agencies often require extensive validation of AI models to ensure their safety and efficacy, which can prolong the timelines for bringing new therapies to market. Establishing clear guidelines and standards for the validation and acceptance of AI models in drug development is crucial to facilitate the integration of these advanced technologies into clinical practice. Early engagement with regulatory bodies during the development process can help address potential concerns and streamline the approval process, fostering a more conducive environment for innovation.

### 5.3 Complexity of biological systems

The multifaceted nature of biological systems and the inherent unpredictability of drug interactions present significant obstacles to successfully applying AI in drug development. AI integration must account for the complexities and nuances of biological processes to achieve reliable and reproducible outcomes. This necessitates interdisciplinary collaboration between AI specialists, biologists, pharmacologists, and clinicians to ensure that predictive models are robust, biologically relevant, and applicable to real-world scenarios. By bridging the gap between computational and biological sciences, researchers can enhance the effectiveness of AI-driven approaches in drug discovery and development.

### 5.4 Ethical considerations

The utilization of AI in drug development raises a myriad of ethical concerns, particularly in relation to data privacy, algorithmic bias, and the potential for unequal access to advanced therapies. Ensuring that AI-driven methodologies are transparent, equitable, and in compliance with ethical standards is critical for fostering public trust and acceptance. Implementing robust safeguards to protect patient data is paramount, alongside initiatives to ensure that AI technologies are accessible to diverse populations. Addressing these ethical issues proactively can help mitigate potential risks and enhance the societal impact of AI in healthcare.

## 6 Conclusion and future directions

**Figure 1:**
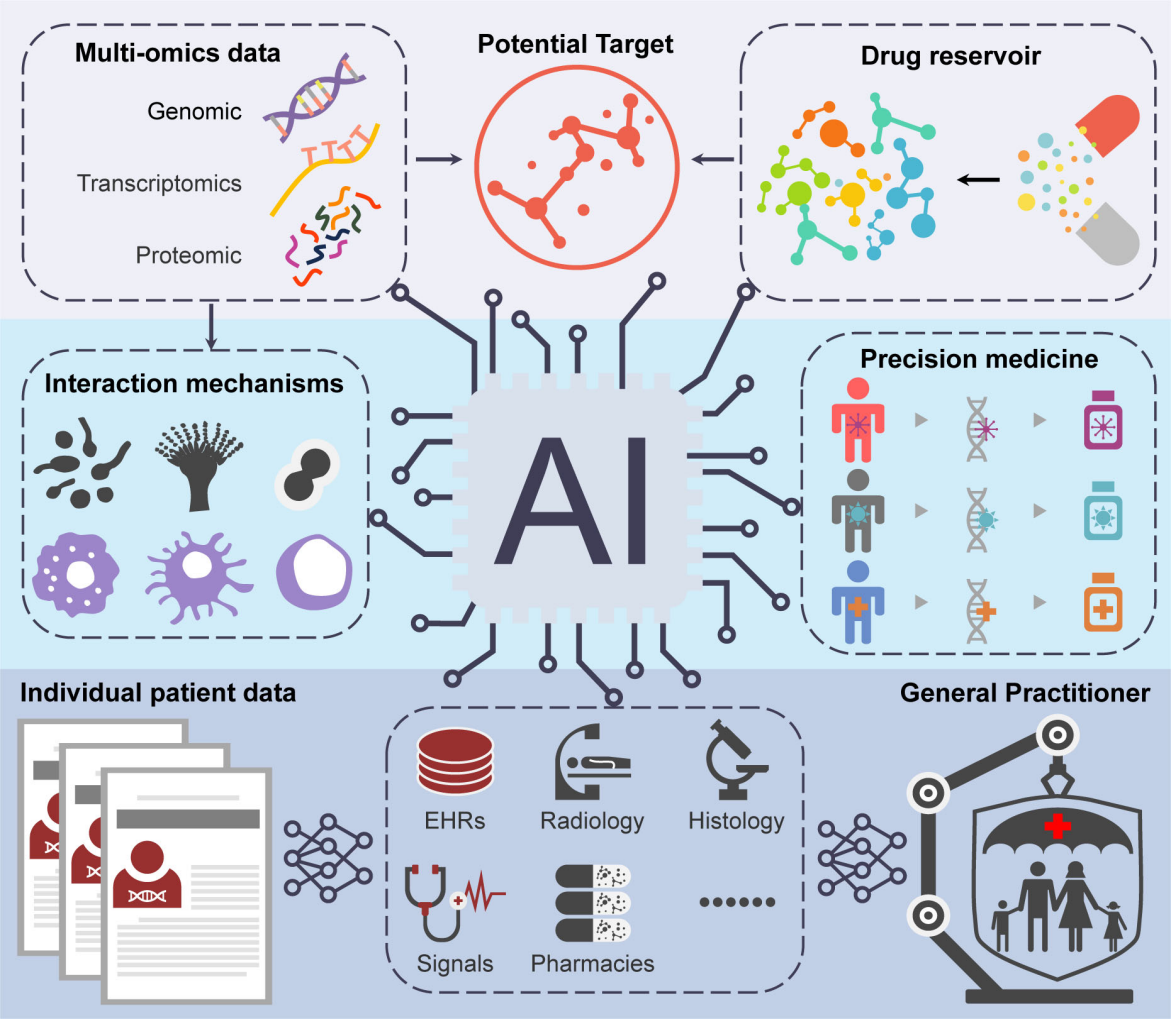
The schematic overview depicting the possible applications of AI in the development of antifungal drugs.

The integration of AI into fungal drug development holds considerable promise for addressing the pressing challenges associated with antifungal resistance and the limitations of existing therapies (**Figure 1**). By enhancing drug discovery processes, optimizing lead compounds, and facilitating personalized treatment approaches, AI can significantly contribute to the development of effective antifungal agents. However, realizing the full potential of AI in this domain will require addressing critical challenges related to data quality, regulatory frameworks, and the complexities of biological systems. Future research should prioritize creating comprehensive datasets, establishing clear validation guidelines for AI models, and fostering interdisciplinary collaboration among researchers, clinicians, and data scientists. Continued advancements in AI technologies, such as natural language processing and reinforcement learning, may open new avenues for innovation in fungal drug development. As we explore the intersection of AI and medicine, it is imperative to remain vigilant regarding ethical considerations to ensure that these technologies are applied equitably and transparently. Ultimately, the successful integration of AI in fungal drug development has the potential to revolutionize our approach to combating fungal infections, improving patient outcomes, and addressing a critical public health challenge. As our understanding of fungal pathogens and AI technologies continues to evolve, the future of antifungal drug development appears increasingly promising, paving the way for novel therapies capable of effectively combating the growing challenge of fungal diseases.
